# Supplementary material for: Content-rich biological network constructed by mining PubMed abstracts
Source: BMC Bioinformatics. 2004 Oct 8;5:147. doi: 10.1186/1471-2105-5-147 (PMC528731; doi:10.1186/1471-2105-5-147)
Supplement: Additional File 5 — The original Chilibot query results of the term "long-term potentiation (LTP)" and 22 other terms, limiting the latest references analyzed to the years 1990, 1995, 2000, and 2004. [file 1471-2105-5-147-S5.bz2 › chilibotAdditionalFile5/ltp1995/html/NMDA_ARC.html]

 


 **NMDA** and **ARC** 
  
Found 7 abstracts in PubMed,  **7 abstracts were retrieved and analyzed**.  


---

 Search Google  |
 PDF files only 
|  EDU domain only 

---

**Interactive relationship** (e.g. stimulation, inhibition, etc)

- The rank order of potency for reduction of  **NMDA**  receptor single channel conductance by polyamines was  **ARC**  greater than SP greater than SD greater than PUT = DET, a rank order similar to that for the inhibitory actions of polyamines in receptor binding assays and for the effects of the antagonists on  **NMDA**  receptor whole cell currents.  Ref: 1378923 Mol Pharmacol, 1992
- The ability of  **NMDA**  to enhance LH release was tested in male rats following infusion into the medial preoptic nucleus MPO, anterior hypothalamic nucleus AHY, ventromedial hypothalamic nucleus VMH, and arcuate nucleus  **ARC** .  Ref: 3062296 Life Sci, 1988

- :-)

  **Parallel relationship** (e.g. studied together, co-existance, homology, etc.)

  - The polyamine antagonists  **ARC** , DET, and PUT produced a voltage dependent reduction in  **NMDA**  receptor whole cell currents and reductions in single channel conductance and average open time, even in the absence of polyamine agonists.  Ref: 1378923 Mol Pharmacol, 1992
  - Plasma LH levels were not affected following microinfusion of  **NMDA**  50 pmole into the AHY, VMH, and  **ARC** .  Ref: 3062296 Life Sci, 1988
  - We conclude that  **NMDA** ... receptors in the CVLM play an important role in the reflex  **arc**  that dilates the airways when hindlimb afferents are stimulated by either muscular contraction or electrical stimulation.  Ref: 7836189 J Appl Physiol, 1994
